# Droplet digital PCR assays for the quantification of brown trout *(Salmo trutta)* and Arctic char *(Salvelinus alpinus)* from environmental DNA collected in the water of mountain lakes

**DOI:** 10.1371/journal.pone.0226638

**Published:** 2019-12-18

**Authors:** Eric Capo, Göran Spong, Sven Norman, Helena Königsson, Pia Bartels, Pär Byström

**Affiliations:** 1 Department of Ecology and Environmental Science, Umeå University, Umeå, Sweden; 2 Molecular Ecology Group, Department of Wildlife, Fish and Environmental Studies, SLU, Umeå, Sweden; University of Iceland, ICELAND

## Abstract

Classical methods for estimating the abundance of fish populations are often both expensive, time-consuming and destructive. Analyses of the environmental DNA (eDNA) present in water samples could alleviate such constraints. Here, we developed protocols to detect and quantify brown trout (*Salmo trutta*) and Arctic char (*Salvelinus alpinus*) populations by applying the droplet digital PCR (ddPCR) method to eDNA molecules extracted from water samples collected in 28 Swedish mountain lakes. Overall, contemporary fish CPUE (catch per unit effort) estimates from standardized survey gill nettings were not correlated to eDNA concentrations for either of the species. In addition, the measured environmental variables (e.g. dissolved organic carbon concentrations, temperature, and pH) appear to not influence water eDNA concentrations of the studied fish species. Detection probabilities via eDNA analysis showed moderate success (less than 70% for both species) while the presence of eDNA from Arctic char (in six lakes) and brown trout (in one lake) was also indicated in lakes where the species were not detected with the gillnetting method. Such findings highlight the limits of one or both methods to reliably detect fish species presence in natural systems. Additional analysis showed that the filtration of water samples through 1.2 μm glass fiber filters and 0.45 μm mixed cellulose ester filters was more efficient in recovering DNA than using 0.22 μm enclosed polyethersulfone filters, probably due to differential efficiencies of DNA extraction. Altogether, this work showed the potentials and limits of the approach for the detection and the quantification of fish abundance in natural systems while providing new insights in the application of the ddPCR method applied to environmental DNA.

## Introduction

Environmental DNA (eDNA) is often considered as a powerful tool to investigate the spatial and temporal distributions of many terrestrial and aquatic organisms, primarily for microbial organisms but with an increasing interest for macro-organisms over the past decade [1–4]. Recently, numerous studies applied molecular methods to natural water samples with the aim to quantify the abundance of fish populations—via molecular quantitative methods—or to assess the diversity and composition of fish communities—via DNA metabarcoding approach—with examples of potentials and limits of this approach [5–7]. The continuous excretions and shedding of cells from living organisms leave trace amounts of their DNA in the water and, in theory, it should be possible to evaluate the presence and/or abundance of species via molecular methods applied to water samples. Recent studies have for instance suggested that fish eDNA concentrations can be successfully correlated with fish numbers or biomass estimates although such studies were mostly performed in controlled conditions [8–10]. In contrast, when applied to natural systems, this approach has rarely highlighted the reliability of such methods [11–13]. The absence of consistency between eDNA concentrations and the true abundance of fish populations may be due to the high variability of parameters inherent to aquatic systems such as lake size, depth and volume, and abiotic and biotic factors influencing eDNA persistence and degradation in the water column (e.g. water retention time, temperature, light, oxygen, pH, salinity, microbial activity, see Hansen et al. [14] for review). Moreover, methodological biases may also affect the quality of the recovered eDNA signal from water samples (e.g. sampling volume, sampling representativeness, filtration methods, DNA extraction’s efficiency, PCR inhibitions or low detection rate using quantitative molecular method). However, if these challenges could be overcome, eDNA analysis would offer an efficient and powerful tool for population monitoring. Indeed, meaningful outcomes from such approaches have already been provided for ecological studies tracking invasion fronts [15–16] or establishing community structures [1, 17].

Traditional methods for estimating absolute fish abundance (e.g. mark-recapture techniques) are often both time-consuming and expensive and are therefore often excluded from monitoring programmes [18]. Alternatives include hydro-acoustic techniques, snorkelling surveys and various counting approaches of fish at specific passages during migrations. However, these methods have limited applicability in most lakes. The most common approach is instead to rely on relative abundance estimation of fish populations using multi-mesh gillnet surveys as it is assumed to be directly proportional to fish density [19–21]. Although relatively simple to perform, standardized multi-mesh gillnetting is logistically difficult to execute in remote areas, destructive and requires ethical permissions. Moreover, relative abundance estimates based on gill net catches are inherently biased due to species and size dependency in catchability as well as dependent on season, weather and abiotic conditions [22]. Although the method is designed to cause as little disturbance and impact on the fish community as possible, it kill parts of the fish population whenever applied. This may be a problem particular in smaller lake ecosystems from both ecological and management perspective. There is also an increasing consensus that monitoring of fish populations should be conducted to minimize fish stress and mortality [23]. Therefore, the estimation of concentrations of eDNA molecules as measures of fish population abundance are foreseen to have the potential to become highly requested cost-efficient and non-destructive methods that may overcome above constraints and short comings. Especially, the highly sensitive droplet digital PCR (ddPCR) method provides tentative and promising advantages over more traditional eDNA approaches (e.g., PCR, qPCR) to become a future management tool for fish abundance estimation [9, 24–27]. This method is based on the partitioning of the PCR reaction mixture into thousands of nano-liters droplets in which individual PCR reactions occur. After PCR amplification, an end-point analysis is performed in each droplet to assess the number of copies of the targeted DNA sequences. This assay is based on the fluorescence than can be generated either by (i) double-stranded DNA binding dyes or by (ii) fluorescently labeled oligonucleotide probes. At present, only few studies applied this method on environmental DNA obtained from water samples e.g., [9, 24], illustrating that the ddPCR approach is powerful to detect and quantify the absolute number of DNA sequences present at low rates minimizing in parallel the potential effects of inhibitors on PCR amplifications as suggested by Capo *et al*. [27]. We hypothesize this approach quantifying DNA molecules at a really low detection limit to be suitable for quantifying fish populations particularly in lakes with low abundance populations.

In the present study, we aimed to test the applicability of an eDNA-based ddPCR approach as a future method to estimate fish population abundance via estimates of eDNA concentrations in water samples applying a large scale approach including 28 Swedish mountain lakes. For comparison with the eDNA analysis, we used the common and standardized multi mesh gill netting approach to estimate fish abundances in lakes containing either sympatric or allopatric populations of brown trout (*Salmo trutta*) and Arctic char (*Salvelinus alpinus*) fish species for which molecular tools are relatively rare for eDNA quantification (but see [13, 27–30]). We expected mountain lakes to be especially suitable for our method calibration due to: 1) Stable size structure and population densities between years and low yearly reproduction causing less within-seasonal variation in biomass of fish [31–32] and eDNA abundance [33] and 2) Low temperature, a parameter that is known to slow down eDNA degradation and thus increase its persistence in the system [34]. Finally, we are aware of the inherent variability in gill net catches independent of fish abundance potentially obscuring relationships between our derived eDNA concentrations and gillnet based fish abundance estimates [35]. Still, we argue that our choice of study system is optimal for relative accurate population estimates based on gillnet catches due to the low structural complexity of mountain lakes which reduces the potential impact of specific gillnet locations for CPUE estimates. Likewise, the relatively small size range of the study species in smaller mountain lakes as well as similar swimming abilities, season dependent activity and size dependent resource use [36] likely further reduces the risk of large discrepancies between our CPUE estimates and actual population abundance´s both within and between lakes.

The specific goal of this project was to test for statistical relationships between CPUE-based fish population abundance estimates from the gillnetting method and eDNA concentrations estimates from the ddPCR method. The following strategy was applied: (i) the use and validation of the specificity of primers and probes designed to amplified DNA from Swedish brown trout (*Salmo trutta*) and Arctic char (*Salvelinus alpinus*) recently published by Capo *et al*. [27] (ii) the quantification of the fish eDNA concentrations from multiple 1 L water samples (i.e. spatial replicates) via the ddPCR method (iii) the tests for correlation with gillnet data collected in parallel and evaluation of the influence of environmental variables on eDNA concentrations (iv) the study the effects of filtration methods on fish eDNA concentrations (1.2 and 0.45 μm circle filters vs 0.22 μm enclosed filters).

## Materials & methods

### Study site and measurements of biological and environmental variables

**Fig 1:**
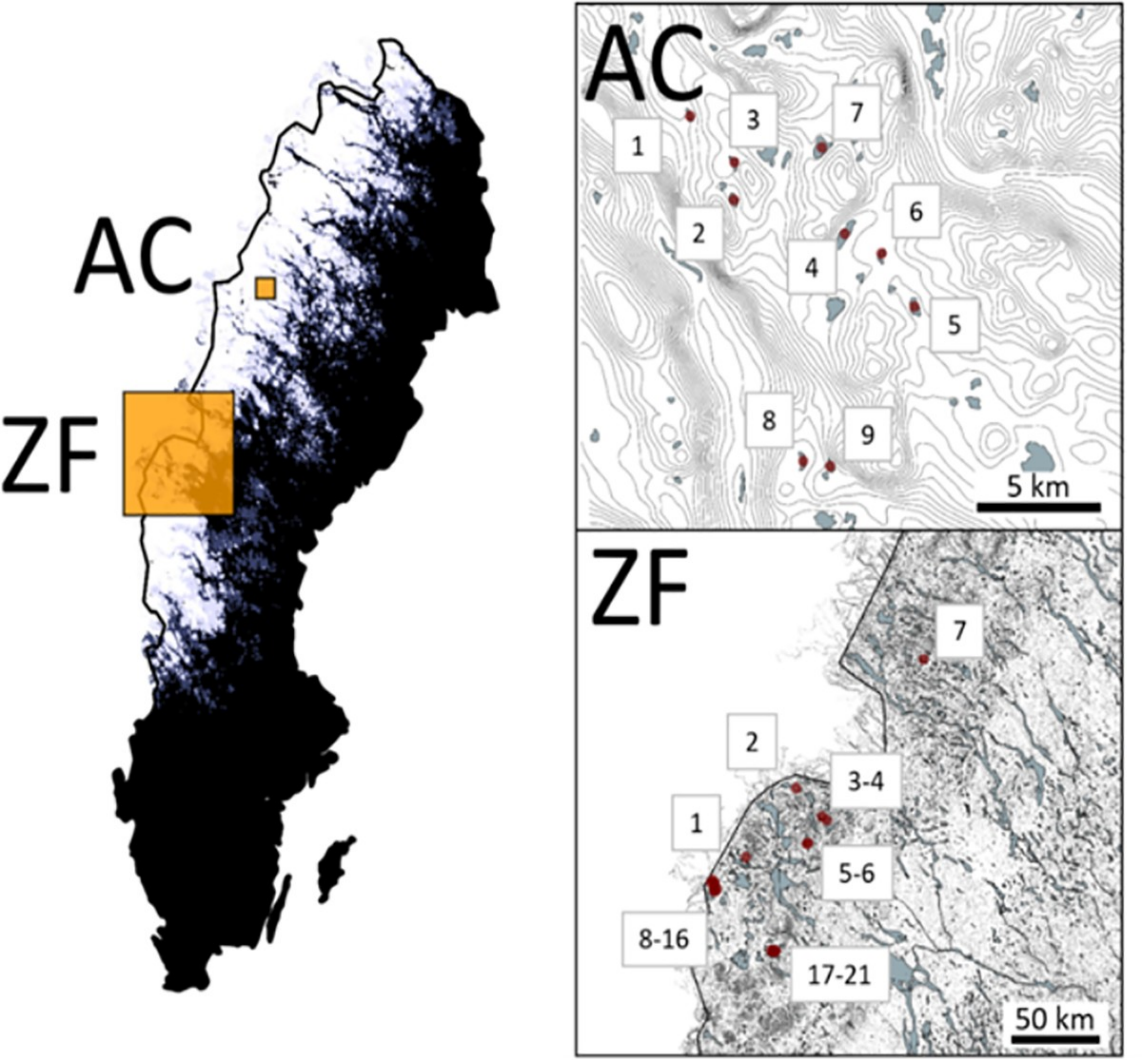
Location of studied lakes from Swedish mountains. The red dots correspond to the position of the sampled lakes.

A total of 28 mountain lakes located in Sweden were selected for the quantification of brown trout and Arctic char populations via molecular methods (Fig 1). Among them, 12 and 7 lakes contain allopatric populations of brown trout and Arctic char (respectively) and 9 lakes contains sympatric populations of both species. The lakes were sampled for fish during summer 2016 and 2017 in accordance with the European standard survey gillnetting method [37]. The method use special made nets (Nordic 12) with mesh sizes varying from 5.5 to 55 mm and take into account lake area and lake bathymetry to determine number and spatial distribution of gillnets set and is designed to representatively catch all species in a specific lake with certain lake area and bathymetry [37]. Thus, the method entails randomized and representable sampling of the whole lake, yielding a whole lake estimate for species occurrence, quantitative relative fish abundance and biomass expressed as Catch-Per-Unit-Effort (CPUE), and size structure of fish communities in temperate lakes based on weighted (by depth zone areal proportion). The obtained CPUE values are the most commonly used measures to infer variation in fish abundance between lakes or between years in the same lake within repeated surveys. In Lakes ZF03, ZF04 and ZF10, the net positions were not recorded and therefore the CPUE values reported could not be weighted by the depth zone areal proportions. Survey gill nettings, methods of sacrifices and design of all fish sampling strategies in this study comply with the current laws of Sweden and were approved by the local ethics committee of the Swedish National Board for Laboratory Animals in Umeå. (CFN, license no. A20-14 to Pär Byström).

Bathymetric parameters were estimated with an echo sounder in the beginning of each field campaign. Lake physical and chemical parameters were measured either continuously (temperature and oxygen profiles) and at approximately monthly intervals (Dissolved Organic Carbon (DOC), Total Dissolved Nitrogen (TDN), pH and the light extinction coefficient Kd) during the ice free period (Table 1). A composite water sample (integrating water collected at 1, 2, 4, 8 m etc.) was taken at the deepest point of each lake for chemical parameters. See [38] for further description of methods regarding the chemical parameters. The light extinction coefficient (*Kd*) was determined from the slope of the linear regression of the natural logarithm of PAR (photosynthetically available radiation) vs. depth. We calculated the parameter Im, a dimensionless integrated estimate of light penetration in the whole water column which incorporates Kd and the average depth and is expressed as a fraction of incoming surface light as described in [39]. Temperature values were obtained from continuous measurements by loggers at a depth of 1 m in the lake during the day of eDNA sampling (except for Lake ZF11 for which a mean of values from 50 and 150 cm was calculated due to lack of logger data at a depth of 100 cm). Mean temperature values were calculated for the day of sampling except for lakes ZF01, ZF05, ZF06 and ZF07 (no logger data available) for which manual measurements of temperature were performed during in early-mid August 2016. We evaluate the stratification or mixing of the water column using the temporal temperature data from loggers.

**Table 1:** Lake typological, physical and chemical parameters.

Lakes	Elevation masl (m)	Area (ha)	Max depth (m)	Mean depth (m)	Light irradiance (Im)	DOC (mg L^-1^)	TDN (μg L^-1^)	pH	Water temperature (°C)	Stratification/Mixing
**AC01**	875	6.85	6	2.7	0.58	1.55	71	8.85	16.13	S
**AC02**	913	13.95	10.8	3.8	0.57	1.24	80	7.95	18.59	S
**AC03**	951	12.17	27.8	10.9	0.42	0.51	47	8.17	14.14	S
**AC04**	899	39.8	4.7	1.8	0.64	1.86	73	7.61	17.77	M
**AC05**	919	20.91	2.9	0.9	0.76	1.66	78	8.07	18.37	M
**AC06**	927	8.28	2.2	0.5	0.89	1.39	72	8.23	18.61	M
**AC07**	998	36.32	22.7	6.7	0.35	0.91	51	8.29	15.17	M
**AC08**	663	12.69	13	2.6	0.5	2.82	105	7.86	18.34	S
**AC09**	708	9.75	11.2	3.5	0.56	2.3	118	8.08	18.16	S
**ZF01**	553	26.38	16.7	4.6	0.49	3.59	97	6.17	11.1	?
**ZF02**	684	5.52	3.5	1.1	0.79	3.78	114	7.14	14.35	M
**ZF03**	821	32.39	13.4	3.8	0.71	1.87	62	7.32	11.89	S
**ZF04**	791	22.71	10.5	3	0.74	1.94	70	6.35	12.49	S
**ZF05**	696	38.51	15.5	3.9	0.69	3.73	96	6.24	13.8	M
**ZF06**	697	22.39	23.1	5.1	0.39	4	100	6.33	13.4	M
**ZF07**	649	18.8	16.3	4.9	0.34	4.11	129	8.36	11.9	M
**ZF08**	501	7.06	14.5	4.8	0.31	3.83	113	6.4	16.84	S
**ZF09**	618	4.02	8.5	2.7	0.35	4.21	118	5.82	15.74	S
**ZF10**	573	13.64	31.2	10.2	0.24	1.95	79	5.84	16.36	S
**ZF11**	564	11.88	14.5	3.9	0.22	5.74	167	5.97	16.43	S
**ZF12**	590	13.37	10.3	3	0.32	4.95	140	5.73	16.21	S
**ZF13**	588	4.27	9.7	3.7	0.19	7.31	171	5.72	15.96	S
**ZF14**	622	4.95	8.7	2.7	0.38	4.7	144	5.66	14.61	S
**ZF15**	576	4.5	3.7	1.3	0.46	6.56	168	5.96	16.17	S
**ZF16**	582	4.68	4.8	1.8	0.35	7.07	176	6.04	16.32	S
**ZF19**	812	9.15	12.7	5.7	0.28	3.12	92	5.98	14.63	S
**ZF20**	840	4.63	7.4	2.7	0.56	2.62	96	6.06	15.12	S
**ZF21**	710	4.78	11.8	3.8	0.29	5.19	147	6.01	16.51	S

### Sampling and filtration of water samples for eDNA analysis

Water samples were collected from 16 lakes in summer 2016 (AC01-AC09 and ZF01-ZF07) and 12 lakes in summer 2017 (ZF08-16 and ZF19-21). For each lake, 1 L of water was collected in sterile Gosselin^™^ HDPE plastic bottles (Fisher Scientific UK Ltd, UK) from five to seven different locations using a Ruttner Water Sampler (KC Denmark). Samples were taken at the deepest location and, depending on lake shape, either in a circular fashion around the deepest location or along one transect for roughly circular and elongated lakes, respectively (see S1 Fig). The 14 sampling controls consisted of 1 L MilliQ water opened bottles during sampling for a subset of the lakes. In 2016 lakes, all samples were taken at ½ max depth for shallow lakes while in deep lakes, two samples were taken at 1 m above the sediment and the remaining samples were taken at 4 m depth. In 2017 lakes, samples were taken at 1 m depth. Furthermore, one sample was taken in the outlet of each lake (both in 2016 and 2017). From all water samples, between 750 mL and 1 L of water were serially filtered through a 1.2 μm glass microfiber filters (Whatman GF/C) and 0.45 μm mixed cellulose ester filters (EMD Millipore) [1.2GF+0.45MCE] using a peristaltic pump. All filters were stored at—20°C until further analyses. Additionally, water samples were also collected at the same locations in all 2017 lakes and filtered using 0.22 μm enclosed filters [0.22GP] (Sterivex) to compare the efficiency of both methods to quantity DNA sequences for each fish species. All filtration equipment was sterilized by soaking for 1 day in 5% bleach and rinsing with 70% ethanol and MilliQ water before and between each filtration, respectively.

### DNA extractions and multiplex ddPCR assays

DNA extraction was performed from filters using DNeasy Blood & Tissue Kit (Qiagen, USA). The DNA was extracted from the 171 1.2 μm glass fiber and 0.45 μm mixed cellulose ester filters that were placed together in the same tube adding 720 μL ATL + 80 μL proteinase K (method [1.2GF+0.45MCE]). The same volume of those products was added to the 58 0.22 μm enclosed filters obtained from water samples (method [0.22GP]). The next steps of the DNA extraction protocol were performed following manufacturer protocols. A total of 13 DNA extraction controls were performed alongside with all processed samples (see S1 Table for correspondence between DNA extracts and their respective DNA extraction controls). The concentration (ng.μL^-1^) and quality (ratio 260/230) of bulk DNA was estimated using a Nanodrop ND-1000 Spectrophotometer (Thermo Scientific, Wilmington, DE, USA).

We performed multiplex ddPCR assays using the primers and probes designed from the cytB mitochondrial region for both species and previously described in Capo *et al*. [27] (Table 2)

**Table 2:** Nucleic acid sequences of primers and probes used for the ddPCR assay (cytB_St1 for brown trout eDNA quantification and cytB_Sa1 for Arctic char eDNA quantification). The TaqMan® probes were composed by FAM and VIC dyes (for brown trout and Arctic char detection respectively), the selected nucleotide sequence and M*GB* (minor groove binder).

	Forward primer 5'-3'	Reverse primer 5'-3'	Probe 5´-3´	bp
**cytB_St1F/1R/pb**	TCCCAGCACCATCTAACATCTCA	ATCTCGGCAAATGTGGCAAACA	VIC-AGGCTTATGTCTAGCCACCCAAATTCTT-MGB	155
**cytB_Sa1F/R/pb**	GACTGCCTTTGTAGGCTACGTT	CAGCGGAGAGGAGGTTTGTG	FAM-GGGCAAATATCCTTCTGAGGAGCCA-MGB	80

Each ddPCR reaction mixture contained 2 μL of DNA template, 200 nM of primers and TaqMan MGB-probe for Arctic char, 400 nM of primers and TaqMan MGB-probe for brown trout, 10 μL of 1× Bio-Rad Supermix for Probes (Bio-Rad, Hercules, CA, USA) with ultra-pure sterilized water up to a total volume of 22 μL. From this reaction mix, 20 μL was mixed with Bio-Rad droplet generator oil and partitioned into up to 20,000 droplets using the Bio-Rad QX-200 droplet generator (Bio-Rad). PCR reactions were performed in sealed 96-well plates with the following conditions: 5 min at 95°C, 40 cycles of denaturation for 30 s at 95°C and extension for 60 s at 62°C, followed by 5 min at 4°C, 5 min at 95°C and a hold at 4°C. After PCR amplification, plates were transferred to the Bio-Rad QX-200 droplet reader (Bio-Rad). PCR optimizations were previously performed to select suitable primers concentration and extension temperature for the amplification of both target. The species specificity of both primer sets and probes was verified using the software Primer-BLAST with default settings [40]. Results show that only online DNA sequences from the same target species have 100% matches. Furthermore, both primers and probes sequences were designed to have a least 2 mismatches with non-target species more particularly between the studied two species of the present study. In situ tests of specificities were apply (via ddPCR assays, see methods below) to DNA extracts from fish tissues from Arctic char and brown trout and showed overall no cross-amplifications [27]. However, while no ddPCR assays amplified DNA from Arctic char tissues with primers and the probe specific for brown trout, a low number of positive droplets (e.g. max at 5 for one replicate) was found in a part of the brown trout tissue´s DNA extracts when PCR-amplified using the primers and probe designed that selectively amplified Arctic char DNA (S1 Table). This phenomena only appeared when ddPCR assays were performed for DNA extracts from fish tissues alongside with DNA extracts from water samples. We suspected a slight cross-contamination between fish tissues or the resulting DNA extracts during handling, a specific amplification being highly unlikely due to the high specificity of the molecular tools (two level of specificities with primers and probes and at least two mismatches in primers and probes). In complement, the specificity of primers to amplify the desired target was verified by cloning-sequencing in DNA extracts from a pair of water samples from mountain lakes as following: PCR reactions were performed in a total volume of 10 μL following the protocol described above. The PCR protocol includes an initial denaturation at 95°C for 15 min followed by 40 cycles of 30 s at 95°C and 1 min at 62°C. The amplicons were then subjected to 5 min at 4°C and 5 min at 90°C. PCR amplicons were cloned using CloneJet PCR cloning kit (ThermoScientific), followed by purification and Sanger sequencing (Eurofins). Sequencing results confirmed the specificity of each primer set.

The ddPCR reactions were run in triplicates for a total number of 256 DNA extracts (229 from water samples, 14 from sampling controls, 13 from DNA extraction controls). The ddPCR reactions that failed amplifying DNA extracts in triplicates were repeated and, if still failing, the samples were discarded from the final dataset. The Bio-Rad’s QuantaSoft software version 1.7.4.0917 was used to quantify the number of copies of target DNA by μL of DNA extract. Each droplet for each well was checked for TaqMan fluorescence to count the number of droplets that yielded positive/negative results. Positive controls—DNA extracts from Arctic char and brown trout tissues diluted at 1/100—were used to define a range of fluorescence to consider positive results and to check for repeatability between ddPCR assays. A lower threshold for a positive signal was arbitrarily defined to increase the stringency level: any droplet beyond the fluorescence threshold was counted as a positive event (2800 for brown trout and 850 for Arctic char). Outliers, i.e. droplets found with fluorescence values far beyond the range of the positive controls, were discarded. The ddPCR reactions with less than accepted 8000 droplets were discarded from the analysis. The raw data are available on S1 Table and the data files on figshare (https://doi.org/10.6084/m9.figshare.9929009.v1).

### Data analysis

False positives in ddPCR assays were represented by one or two droplets detected in the fluorescence range of positive controls in a part of controls (S1 Table). However, it was never detected in triplicates ddPCR assays and were therefore considered as random noise and discarded with the following procedure: (i) ddPCR reactions showing less than 3 droplets were considered as negative (0 droplet) (ii) only samples for which subsequent positive droplets were found in a least two of the three replicates were considered as positive (iii) for remaining samples thus considered as positive, mean, median, minimum and maximum values of positive droplets were calculated. Then, for each sample, trout and char eDNA concentrations were calculated per DNA extract by dividing the mean number of positive droplets by the volume used in ddPCR reaction (1.8 μL) and multiply by the total volume of the DNA extract (100 μL) (S2 Table). Additionally, a mean value of eDNA concentration was calculated for each lake. The ddPCR results from the DNA extracts from Lake ZF13 were discarded due to a relatively high amount of positive droplets for brown trout DNA (97 up to 12329 copies) detected in the DNA extraction control extracted alongside with ZF13 samples (i.e., DNA extraction control number 11, S2 Table).

To identify the factors that may influence fish eDNA concentrations (mean, median, minimum and maximum values) in the studied lakes, we used both linear, exponential (lm function) and generalized linear mixed models (GLMM). For GLMM modelling, we used R scripts from Harper et al. [41] to test different models using the functions *glmer*, *glmer*.*nb*, *glmmadmb* and *glmmTMB* (R packages lme4, glmmADMB, glmmTMB respectively, [42–44]). The collinearity of all environmental and technical variables was assessed using Spearman’s correlation coefficient and variance inflation factors (vif function from R package car; [45]). Variables were considered as collinear if Spearman r > 0.3 and VIF > 3. The GLMM modelling was performed independently for both species using the trout and char eDNA concentration (both non-transformed and log-transformed values) for the response variables. Lakes was modelled as a random effect in the model. Predictor variables were centered and scaled to have a mean of 0 and a standard deviation of 1. Validation checks were performed to detect the model that fit the best for the two datasets. Model fits were assessed using the Hosmer and Lemeshow Goodness of Fit Test [46] using the R package ResourceSelection [47]. A total of 28 models was tested for each response variable. R scripts and outputs of GLMM analysis are described in details in the S1 File.

## Results

### DNA extraction efficiencies from water samples

**Fig 2:**
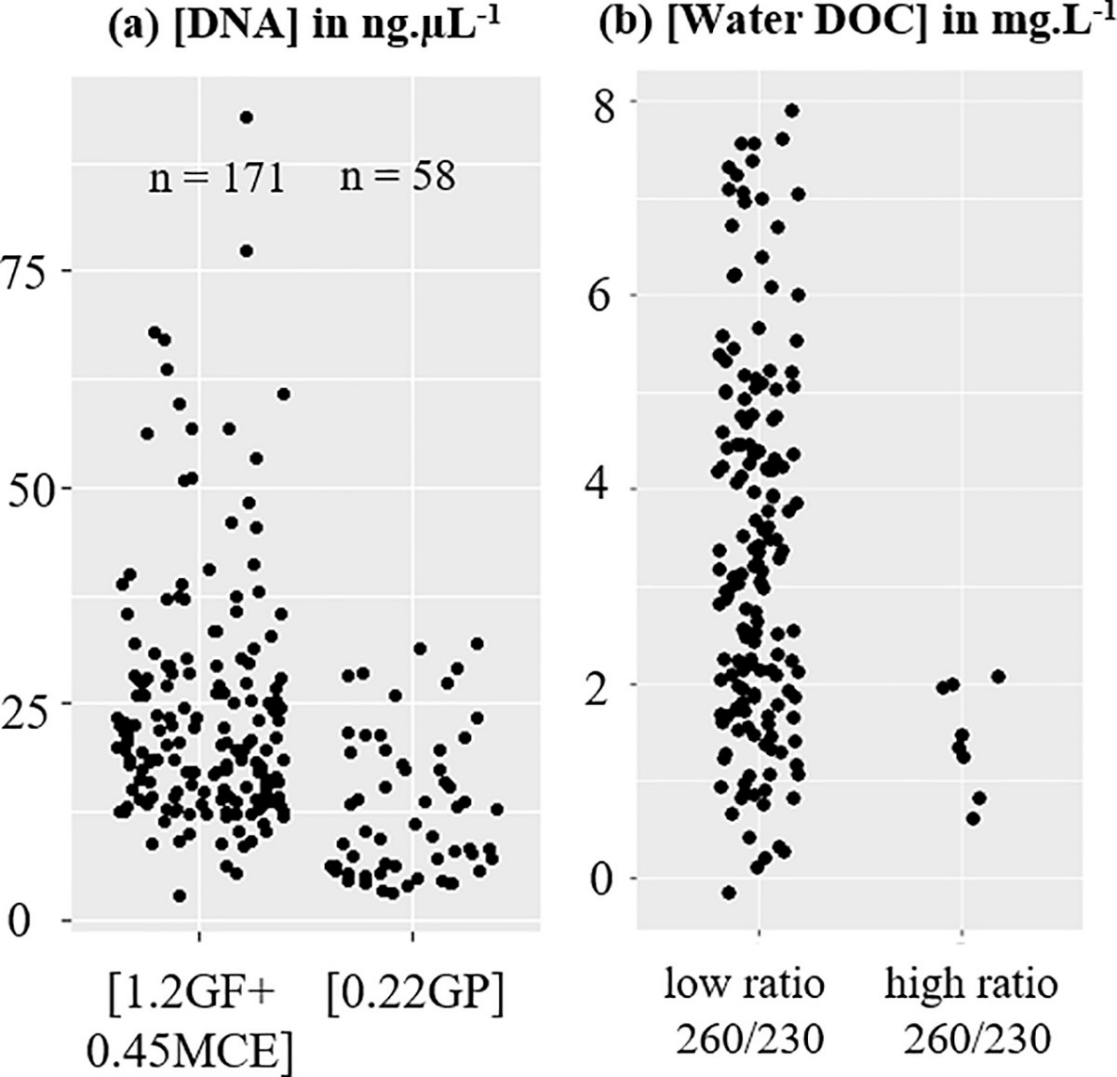
DNA extraction efficiency. (a) DNA concentrations measured (in ng.μL^-1^) using the two filtration methods ([1.2GF+0.45MCE] and [0.22GP]) (b) Relationships between quality ratio of DNA extracts (ratio 260/230) and concentration of water DOC from each lake.

The concentrations of the 171 DNA extracts obtained from serial filtrations with 1.2 and 0.45 μm filters [1.2GF+0.45MCE] ranged from 2.9 to 93 ng.μL^-1^ (mean 24.3 ng.μL^-1^) (Fig 2A). To evaluate the quality of the DNA extracts, we used the 260/230 ratio calculated from the absorbance values obtained by Nanodrop measurements. Low 260/230 ratios (< 0.95) indicate the co-extraction of compounds (e.g., humic substances) with DNA molecules that may hamper PCR amplifications. Most of DNA extracts from [1.2GF+0.45MCE] filters showed low 260/230 ratios (94% of samples with ratios < 0.95) and low ratios were found in samples from lakes with low and high DOC (Dissolved Organic Carbon) values. In contrast, DNA extracts with high ratios (> 0.95) were only obtained from lakes with low DOC concentrations (Fig 2B). The 58 DNA extracts from 0.22 μm filters [GP] showed lower DNA concentrations from 3.2 to 32 with a mean around 12.8 ng.μL^-1^ (Fig 2A). In addition, only 3 DNA extracts from [0.22GP] filters showed positive amplifications of fish eDNA: 2 samples in Lake ZF08 for Arctic char and 1 sample in Lake ZF14 for brown trout, see S2 Table). Therefore, the ddPCR results from this type of filters was not used for further analysis.

### Detection of fish eDNA in water samples from mountain lakes

**Fig 3:**
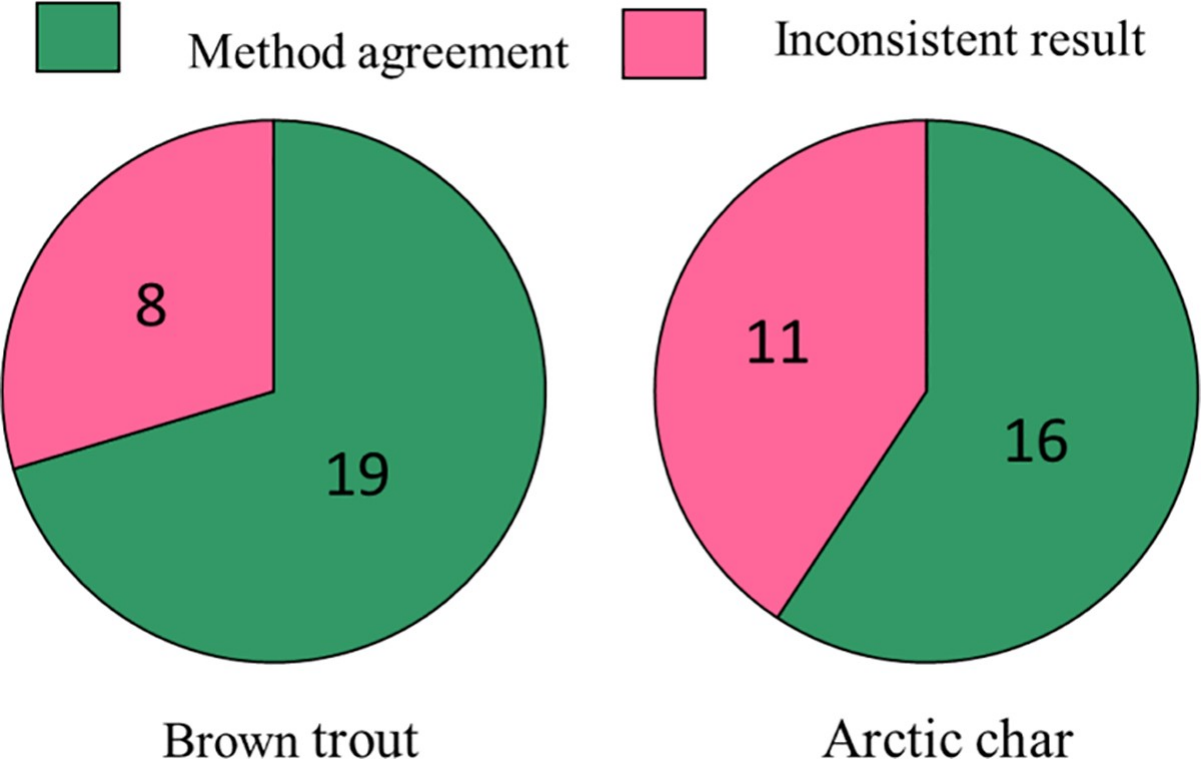
**Number of lakes in which method agreement (gillnet vs eDNA) were found (in green) or not (in pink).** Method agreement include lakes in which fish were found either present or absent with both methods. Inconsistent results corresponds to lakes in which fish were detected with the gillnet method but not via the eDNA method and vice-versa. No comparisons were performed for one lake due to the lack of eDNA quantification (i.e. ZF13) resulting in a total of 27 lakes.

The eDNA detection rate of fish species showed only moderate efficiency when compared with the results of the gillnetting method (Fig 3, Table 3). For brown trout, method agreement was found for 19 lakes corresponding to 70% of the total number of lakes. For the eight lakes showing inconsistent results, only one lake had positive eDNA record–but for only one of the six spatial replicates–when no trout was caught with the gillnetting method (Table 3). The seven remaining lakes were characterized by detection with the gillnet method while no eDNA detection. For Arctic char, only 60% of the lakes (i.e. 16 lakes) showed method agreement while char eDNA was recorded in a relatively high number of lakes (6 lakes) where no char were captured with the gillnetting approach.

**Table 3:** CPUE estimates and mean eDNA concentrations for each lake for brown trout and Arctic char species. The eDNA concentrations described are mean values calculated from spatial replicates from each lake. The number of positive spatial replicates is also displayed. Sampling time (YY-MM-DD) are displayed in this table.

Lakes	Sampling time		Brown trout			Arctic char		
	Gillnetting method	Molecular method	CPUE estimates	eDNA concentrations		CPUE estimates	eDNA concentrations	
				all samples	positive replicates		all samples	positive replicates
**AC01**	160801	160728	0	80	1/6	706	0	0/6
**AC02**	160802	160728	358	37	1/7	0	0	0/7
**AC03**	160803	160728	0	0	0/4	484	0	0/4
**AC04**	160817	160726	1278	0	0/4	0	0	0/4
**AC05**	160819	160726	2196	0	0/5	0	0	0/5
**AC06**	160818	160726	1043	77	1/6	0	0	0/6
**AC07**	160816	160726	0	0	0/5	1222	607	3/5
**AC08**	160805	160727	1611	132	2/7	45	79	2/7
**AC09**	160805	160727	1615	0	0/4	241	324	1/4
**ZF01**	160727	160712	657	19	1/6	254	275	5/6
**ZF02**	160816	160713	959	40	1/6	0	52	2/6
**ZF03**	160831	160715	0	0	0/6	51	74	2/6
**ZF04**	160820	160715	0	0	0/6	141	83	1/6
**ZF05**	160809	160714	282	77	3/6	634	5685	5/6
**ZF06**	160810	160714	135	0	0/6	329	296	4/6
**ZF07**	160823	160717	1337	83	2/4	0	3801	2/4
**ZF08**	170729	170807	224	90	3/6	148	179	4/6
**ZF09**	170730	170805	305	56	2/6	0	59	2/6
**ZF10**	170727	170806	197	0	0/6	97	0	0/6
**ZF11**	170728	170806	680	28	1/6	476	22	1/6
**ZF12**	170726	170803	491	93	2/6	258	0	0/6
**ZF13**	170724	170805	395	-	-	0	-	-
**ZF14**	170725	170803	336	119	3/5	0	0	0/5
**ZF15**	170724	170804	550	80	1/6	0	231	5/6
**ZF16**	170725	170804	518	0	0/4	0	97	2/4
**ZF19**	170802	170801	0	0	0/6	507	28	1/6
**ZF20**	170802	170801	0	0	0/6	2113	0	0/6
**ZF21**	170801	170802	313	0	0/4	0	398	1/4

### No relationships between eDNA concentrations, CPUE estimates and environmental/technical variables

The mean eDNA concentration for Arctic char varied from zero to 5685 DNA copies with a mean value of 478 DNA copies per sample (Table 3, S2 Table). The highest copy number was found in Lake ZF05 in which the Arctic char CPUE estimate (i.e. proxy of the relative abundance of fish populations) was relatively high (i.e. 634 g of char per net). The mean DNA copy number for brown trout was lower (46 DNA copies) with the highest DNA copy number at 132 (Lake AC08) in which the brown trout CPUE estimate was similarly relatively high with a catch of 1611 g of trout per net.

**Fig 4:**
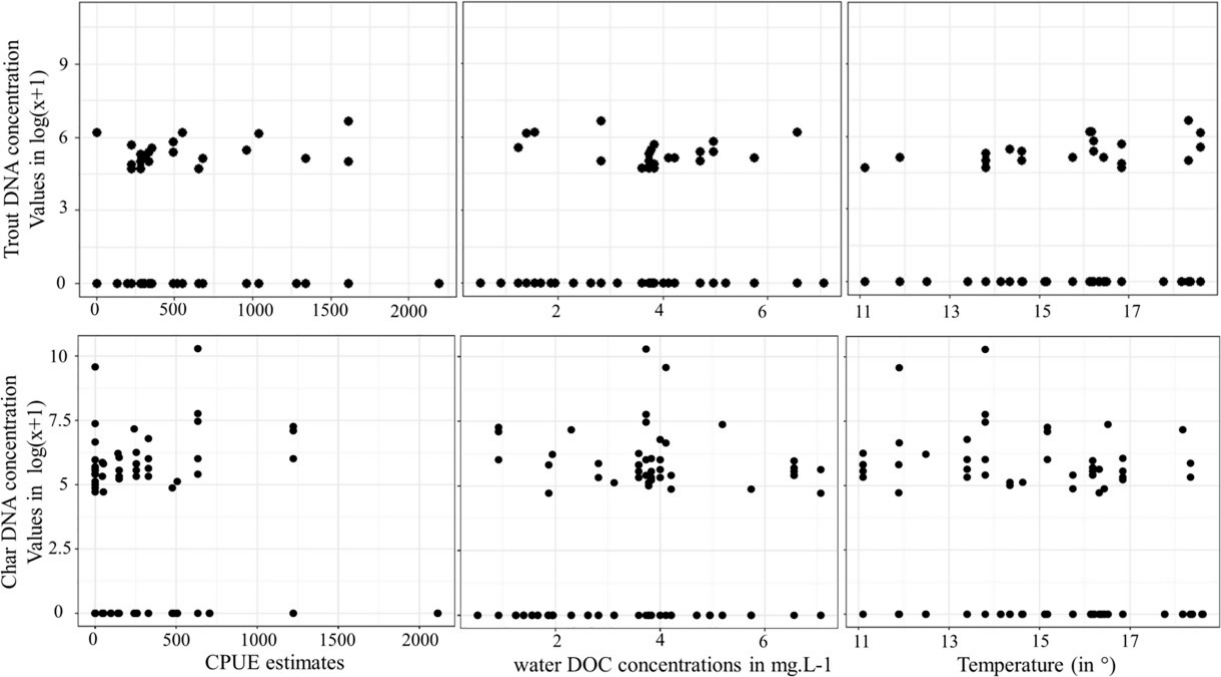
Relationships between the log-transformed eDNA concentrations (in copy number) and CPU estimates, DOC and temperature values for each species.

To evaluate which parameters influence the eDNA concentration in water, we used CPUE estimates and environmental parameters that may induce in situ degradation of DNA molecules via abiotic or biotic processes (DOC, temperature, TDN, Kd, Im, pH, see Fig 4). We also consider the technical parameters that can hamper eDNA concentrations (filtered water volume, DNA concentrations and DNA quality (ratio 260/230)). The variables TDN, Kd, Im, pH, filtered water volume were found collinear with other parameters and removed from the model analysis. CPUE, DOC, Temperature, DNA concentrations and ratio 260/230 were kept as explanatory variables (CPUE estimates from Arctic char and brown trout respectively from both models). Linear and exponential models did not satisfy the necessary assumptions (normality and homoscedasticity). In addition, most of GLMM models did not satisfy the validation checks (normality of data and residuals). However, for both response variables, a zero-inflated Poisson distribution model was identified as the best fit (with glmmTMB function) and this model did not detect any influence of selected factors on mean, median, minimum and maximum values eDNA concentrations of trout and char as shown in Supplementary Material (S1 File).

## Discussion

We aimed to evaluate the use of the ddPCR approach for the quantification of species-specific DNA molecules retrieved from lake water sample with the goal to estimate the abundance of brown trout (*Salmo trutta*) and Arctic char (*Salvelinus alpinus*) populations. Using an extensive large scale approach that includes 28 lakes, no relationships were found between standard fish populations estimates (i.e. CPUE estimates) and eDNA concentrations of either species. Under the assumption that the gillnet method is a reliable method for the estimation of the abundance of fish populations, our findings corroborate other studies aiming to quantify fish populations from eDNA water samples e.g., [14, 33]. Our results confirm the limits and challenges of applying this approach in water from natural systems—in comparison with controlled experiments—and highlights that the natural variability in lake physics, chemistry and biology may unpredictably disrupt the direct correlation between fish abundance and eDNA. Altogether, our results highlight the challenge and present constraints of utilizing the eDNA approach as a stand-alone method for fish surveys, for both detection and abundance estimates.

In our study, the detection rates of both species via the eDNA approach, when compared to the gillnetting method, showed moderate success with less than 70% lakes with species detected with both methods (Fig 3). Although these detection probabilities can be considered sufficient in an eDNA context, they can be considered low in comparison to standard gillnetting methods because even a few nets are highly likely to catch most present species especially in relatively small and species poor lakes [48]. Degerman et al [48] for instance showed that on average only 2.2 and 5.3 multi mesh gillnets are needed to capture all species in 10 ha respectively 100 ha large lakes. Hence, our effort of 8–16 gillnets used in our study lakes with a size range 4–40 ha are thus substantially higher, suggesting that our conclusion of presence absence of Arctic char and brown trout based on gillnets catches are valid. Still, due to limits in the detection sensitivity of some fish species by gillnetting method, such as e.g. for eel (*Anguilla Anguilla*, [48]), traditional monitoring may not be suitable to use for all species as a baseline to compare with eDNA detection rates. In our study, as net distributions in lakes includes a larger spatial coverage than our eDNA sampling we cannot rule out the possibility that a similar spatial coverage of eDNA samples could have provided significant correlations between respective abundance estimates obtained from both methods. The detection probability decreasing with distance from the fish (as tested with caged northern pike in lake water by Dunker et al [49]), our spatial distribution of eDNA samples did not covered near shore areas in which fish abundance may be the highest [32]. Fish DNA molecules were also detected in water samples from six lakes were no char were caught in gillnets, while for brown trout, such discrepancy was only present in one spatial replicate from one lake (AC01). While we cannot rule out the possibility that we failed to detect species presence with our gillnet approach, this is at odds with present and historical knowledge of local fishermen and fishing right owners of species presence in our study lakes. Furthermore, we have carefully checked for the possibility that above or below lake streams could have provided either input of lake water eDNA of char from upstream lakes with char or that occasionally downstream or upstream migrations of a few char individuals may occur. Neither of the possibilities were considered likely based on connectivity analysis on detailed map and satellite images except for ZF21 from which char or eDNA molecules may have migrate from the lake ZF20 located upstream. Another factor that could explain such differences between the two methods is the presence of allochthonous eDNA transported by e.g., water birds, into the studied lakes as such molecules previously found to be detectable a least one month after their deposition [50–51].

In addition, the occurrence of true false positives (i.e., methodological issues) can be caused by many factors including (i) contamination of lake water as the same nets were used in all lakes, (ii) contamination of samples during DNA extractions and subsequent analysis and (iii) an nonspecific amplification by primers and probes designed to quantify Arctic char DNA. (i) Nets were used one or two weeks after eDNA sampling during the 2016 field campaign, and cross-contamination of eDNA between lakes by nets was not possible for lakes AC01-09 and ZF01-07. However, in 2017, the nets were used a day or up to a week prior to eDNA sampling (ZF08-16, ZF19-21) (Table 3). Therefore, the possibility of eDNA cross-contamination during the field work is one potential explanation for the detection of Arctic char eDNA in lakes where char was not present based on our gillnetting results. Still, we argue that this explanation seems less plausible because such cross contamination should have been detected for brown trout DNA also but we did not find a similar possible contamination pattern in samples from lakes with no trout. (ii) No contamination was detected in DNA extraction controls (except the one extracted alongside with ZF13 samples as described in the Material and Methods section), and we therefore do not suspect cross-contamination during DNA extraction while still considering that it maybe one explanation of the presence of those false positives. The presence of false positives may also have been caused by a methodological issue related to the ddPCR procedure. More specifically, users recently suggested that too short incubation time at low temperatures—4 to 12°C—of 96 well plates after PCR and prior to processing through the QX200 reader may increase the presence of false positives (i.e. positive droplets, unpublished data). (iii) As described in Material and Methods, the primers and probes were designed carefully and validated by in silico test, in vitro PCR tests on tissues both fish species and cloning-sequencing of PCR products from lake water samples. These tests allow us to be assure about the specificities of the primers sets and probes designed in this study. However, a weak PCR amplification of brown trout DNA by Arctic char species-specific primers and probe could be one explanation of the detection of Arctic char eDNA in water samples from lakes where only brown trout was detected with nets. We thus recommend to test the in vitro specificity of the Arctic char molecular tools set before application of qPCR/ddPCR assays on DNA extract from complex natural systems. Altogether, despite thorough retro-perspective analysis of field and laboratory procedures in the light of above, we have not been able to identify factors explaining the inconsistency of results between the gillnet and eDNA methods.

The reliability of eDNA concentration estimate to quantify fish population abundances is under debate among molecular ecologists (see [14, 33] for review about potentials and limits, [52–53]). While some studies have showed that species-specific eDNA concentrations are positively correlated with biomass and abundance estimates such as [8, 10, 29, 34, 54], others showed the absence of such relationships [13, 55–56]. In our study, eDNA concentrations were not found to be correlated with CPUE estimates highlighting the non-reliability of the eDNA method to quantify fish abundance in natural systems with the methodology applied in the present study. Still, it is important to note that this conclusion rest on the assumption that the CPUE estimates of abundance are reasonably accurate. We acknowledge that gill net catches are inherently variable due to many factors e.g. weather condition, time of season and lake morphology. However, the followed protocol for survey gill netting [37] take into account lake morphometry and is standardized for season. This combined with the low structural complexity in mountain lakes in general—and in our sampled small mountain lakes in particular—and generally stable weather conditions during our samplings suggest that the uncertainties in the CPUE estimates are likely minor (Norman unpublished data). Correspondingly, the work of Deutschmann et al. [13] focusing on brown trout DNA from ponds with different biomass found no correlation with total fish biomass, and authors suggested that size and life stage differences in fish populations may offset relationships between biomass and eDNA concentrations. In contrast, Klobucar et al. [29] showed strong correlations between Arctic char eDNA concentration and fish biomass in five northern Alaska lakes from Alaska. The authors suggested that their success to quantify fish populations may be related to the fact that they targeted small lakes closed to fish emigration and immigration and with simple fish communities (only two species), conditions we also took into account in the present work. Nevertheless, in our study, the detection and quantification of fish populations by the eDNA approach may have been hampered by, in some cases, low density of fish populations as well as larger size range of the lakes investigated (here from 4 to 40 ha; from 0.7 to 10.9 in [29]). Following findings from Klobucar et al. [29], we also investigated if eDNA concentrations were found higher in samples from deep waters than in samples from shallow waters in relationships with mixing or stratification of the water column (depending of lake, see Table 1) but no clear relationship was found.

As stated in Hansen et al. [14], the quantity of eDNA molecules in water samples appear to be controlled by three processes: the production rate from the target fish species (e.g. shedding rates of eDNA), the physical transport of DNA molecules and the degradation rate of the DNA molecules due to abiotic and biotic processes. As debated over the recent years, shedding rates of eDNA from fish individuals are still unclear, even when investigate in controlled experiments [10, 52] and appear to be related to body mass i.e. relatively higher for small than large individuals [57]. In natural systems, such parameters may not be easy to quantify precisely and hamper the comparison between biomass estimates and eDNA concentrations. The physical transport of DNA molecules in natural lakes is also likely hard to account for because it is dependent of different processes occurring at various spatial and temporal scales (diffusion, advection, sedimentation, resuspension, Hansen et al. [14]). The measurement of environmental variables in parallel with gill net catches allowed us to investigate what factors may have influenced the fish eDNA concentrations via direct effects on DNA molecules. Due to collinearity with other measured environmental variables as well as their known effect on eDNA detection and/or persistence in natural systems, only temperature and DOC were considered in the models used. While temperature was not identified in our study as a factor correlated to eDNA concentrations, it has been suggested to increase shedding rates of DNA via increased metabolic activity of fish populations while, with antagonistic effects, influencing DNA molecules breakdown in the water column [41, 58–59]. DOC concentration is also known to have impact on fish population abundance, resource and habitat use in lakes [38–39] and thus also potentially the spatial variability of DNA molecules. Altogether, we found no consistent relationships with environmental variables that could have influence the eDNA concentration in our systems probably due to the many factors that may have hampered the presence and the quantity of eDNA molecules in the collected water samples.

While we carefully designed our experimental procedure, based on available literature, to ensure the authenticity of the DNA signal obtained and to increase our chance to properly quantify the desired target, we were unable to establish relationships between eDNA concentrations and CPUE estimates in the studied lakes. Alternative procedures may have been more suitable and successful than ours. For instance, the volume of sampled water for filtration (approximately 1 L) may be a critical issue in our study for detection probabilities of the fish species. While most of studies aimed to quantify eDNA concentrations from 1 L water samples [13, 34, 54, 60], many papers discussed the need of larger volumes to increase detection probabilities of fish species from natural systems [29, 61]. Klobucar et al. [29] collected 5 L samples from their lakes and estimate that even larger sampling volumes would be needed to achieve near 100% detection probabilities in their lakes (> 25 L). Such procedure may be feasible in controlled research projects but is often not feasible in regular monitoring situations particularly in the case for remote mountain lakes. Because fish populations also are inherently unevenly distributed in lakes, a key component to take into account is the use of spatial replicates distributed over the lake area in our sampling design. Indeed, we chose here to apply a large scale approach (i.e. 28 lakes) with a relatively low sampling volume but with spatial replication within each lake (in average, six samples per lake plus one in the outlet; S1 Table). Our results with large variability in detection and concentration of eDNA molecules from spatial replicates from each lake is in line with the potential heterogeneity of eDNA distribution in lakes related to the unequal distribution of fish, variability in eDNA molecules transports and DNA degradation rates. For some lakes, composite samples (vertical transect of the water column) were also collected in order to check for vertical distribution of eDNA molecules, as suggested in Eichmiller et al. [34], but those samples did not show any higher amount of eDNA molecules (S2 Table). One better strategy that may have been applied is the use of more replicates according to the size and volume of each lake. In complement, we suggest in line with the recent findings of Hunter et al. [26] that a more suitable approach to increase fish detection rates is to filter a higher sampling volume through multiple filters at each location. The choice of filtration methods is known to influence the efficiency to capture eDNA [59–60, 62]. In our study, the two selected filtration methods showed strong differences notably due to only few positive results obtained from 0.22 μm filters [0.22GP] compared to DNA extracts obtained from 1.2 and 0.45 μm filters [1.2GF+0.45MCE]. While [0.22GP] filters have been suggested to be strongly efficient to catch fish DNA molecules [60, 63] and specifically useful for sampling in remote areas, our results showed smaller concentrations of DNA and poor quality (ratio 260/230) compared to [1.2GF+0.45MCE] filters. We suspect that, even if such filters caught at least the same quantity of fish DNA molecules than the other method used here, the DNA extraction with DNeasy Blood and Tissue kit was less efficient with the [0.22GP] filters, even applying the same procedure that Spens et al. [2017]. Differences in eDNA quantification may have also been caused by the co-extraction of molecules acting as PCR inhibitors (e.g. DOC, humic and fulvic acids) and thus that may have hampered the PCR amplification. The application of droplet digital PCR (ddPCR) to quantify fish populations from water samples is still rarely used [9, 24–27]. As highlighted by the work of Doi *et al*. [9, 24], the ddPCR shows consistent results with qPCR and can even outperforms the latter for detection of rare fish DNA molecules. Moreover, this method appear to be more powerful because, alongside with DNA molecules, PCR inhibitors are also partitioned with the droplet generation reducing thus the inhibition of PCR reactions by compounds co-extracted with DNA (as it is the case from lake water with relatively high DOC). However, the advantages to be able to detect DNA molecules present at very low rates may also be a constraint, as this method being consequently highly prone to contamination even by very rare DNA from other sources. Correspondingly, we detected low amounts of sequences (i.e. 1 or 2 positive droplets) in controls (sampling and extraction controls) that would not be detected with PCR or qPCR. Thus, our large scale analysis (more than 200 DNA extracts analyzed) showed the need to be careful when interpreting the ddPCR results due to the issues related to false positives. The need to characterize thresholds for positive/negative amplifications is one challenge for the future research aiming to detect low abundant aquatic species from water samples via the ddPCR method.

## Conclusions

This work illustrates that, under the assumption that traditional CPUE estimates of fish population abundance represents true abundance in the study lakes, the eDNA-based ddPCR approach we applied may not be adequate for the detection and quantification of lake living brown trout and Arctic char populations. While the droplet digital PCR method allows to detect DNA molecules at very low concentrations, and thus in theory more powerful for detecting rare species, our results suggests that local lake-specific factors difficult to quantify may prevent the blanket use of eDNA concentrations across lakes as a proxy for fish abundance. Thus, before that the eDNA-based ddPCR is considered a reliable monitoring tool of fish abundance, more studies should be conducted on fundamental aspects related to the fate of fish eDNA molecules in natural systems such as shedding rates of DNA from fish and its physical transport in lakes towards a better understanding of eDNA transport and preservation in aquatic systems.

## Supporting information

S1 Fig Depth maps of each lake. Locations of spatial replicate from in-lakes and outlet samples are displayed by blue and red circles respectively. Yellow circles showed the locations of nets for the gillnetting method. Locations for DNA sampling in 2016 field campaign were not recorded and performed accordingly to the description in the Materials and Methods section.
(PDF)

S1 Table Raw outputs from the ddPCR assays. This table shows the results from the triplicates ddPCR assays performed from each DNA extract originated from water samples, sampling controls, DNA extraction controls and the positive controls (Ste1 for brown trout’s tissue, Sae1 for Arctic char’s tissue). The results of the ddPCR controls are included in this table. For each assay, the number of positive droplets detected for brown trout and Arctic char and the total number of accepted droplets is shown. Background information included lake names and associated CPUE estimates for each species, sample’s id, filter’s types, filtered volume, DNA extraction and ddPCR batches and the concentrations of DNA extracts (DNA_C in ng.μL^-1^). Samples entitled -LO correspond to outlet samples.
(XLSX)

S2 Table Outputs from the ddPCR assays for each water sample filtered with [GF+MCE] filters. This table shows the concentrations of brown trout and Arctic char eDNA molecules found in each DNA extract (mean, median, minimum and maximum values for each sample). Background information included, lake id, temperature and DOC values measured from water samples, CPUE estimates obtained for all lakes and for each species, sample’s id, filtered volume and the concentrations of DNA (in ng.μL^-1^). Samples entitled -LO correspond to outlet samples.
(XLSX)

S1 File R scripts and outputs of GLMM analysis. (R)
