# Supplementary figures and images for: Droplet digital PCR assays for the quantification of brown trout (Salmo trutta) and Arctic char (Salvelinus alpinus) from environmental DNA collected in the water of mountain lakes
Source: PLoS One. 2019 Dec 18;14(12):e0226638. doi: 10.1371/journal.pone.0226638 (PMC6919618; doi:10.1371/journal.pone.0226638)

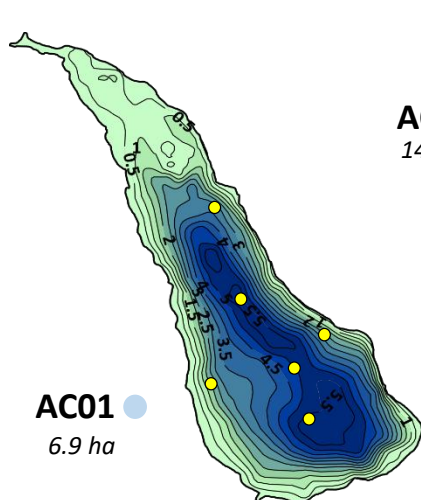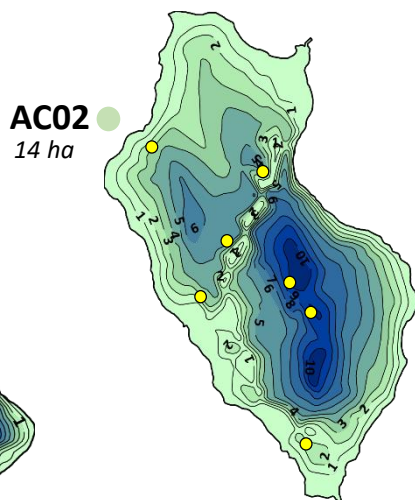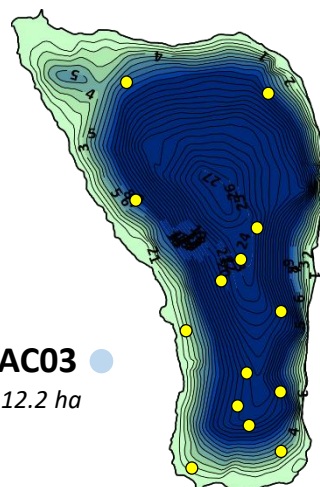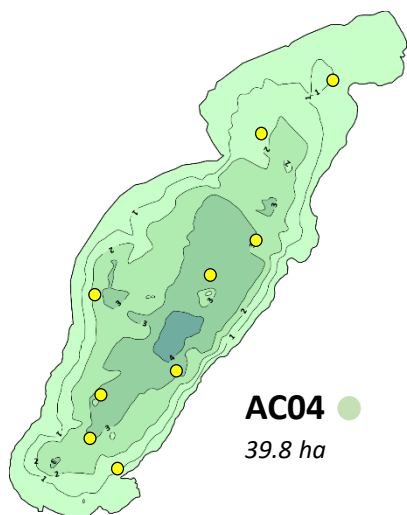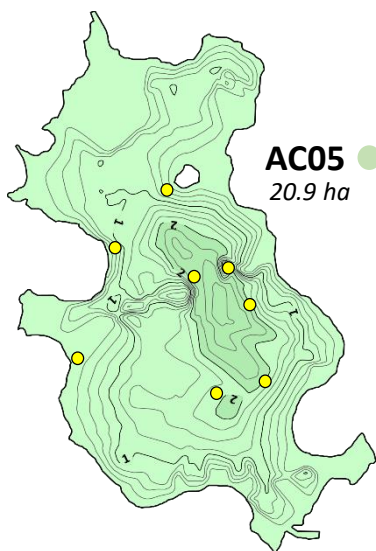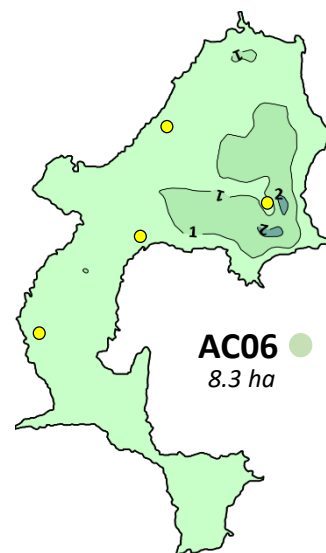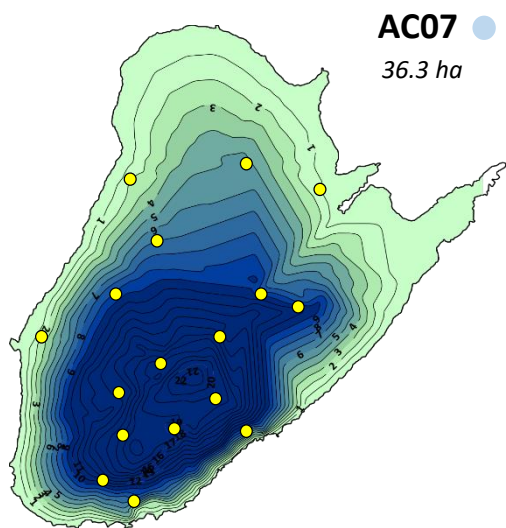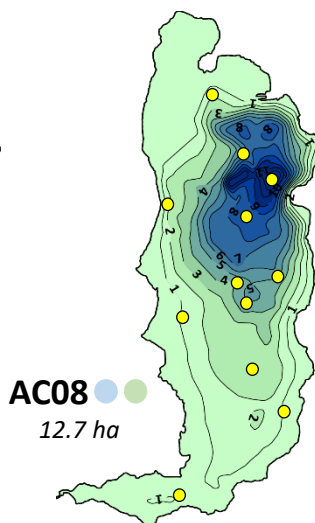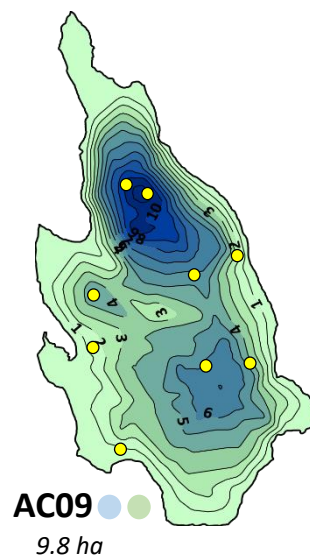

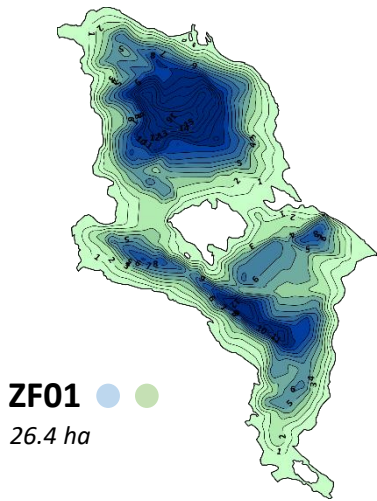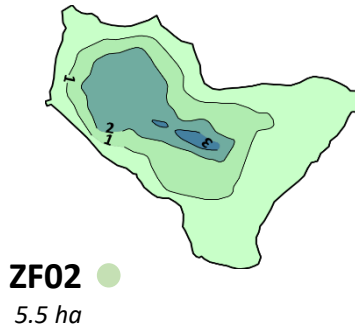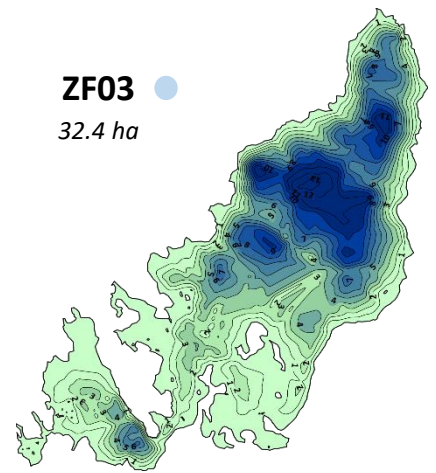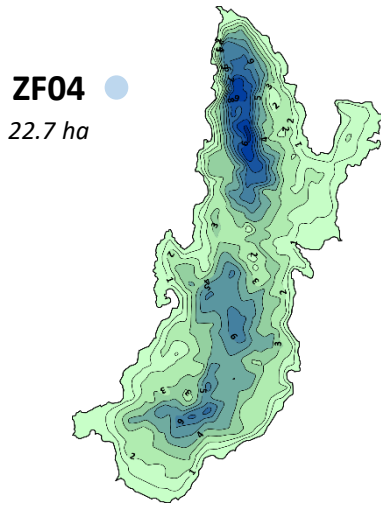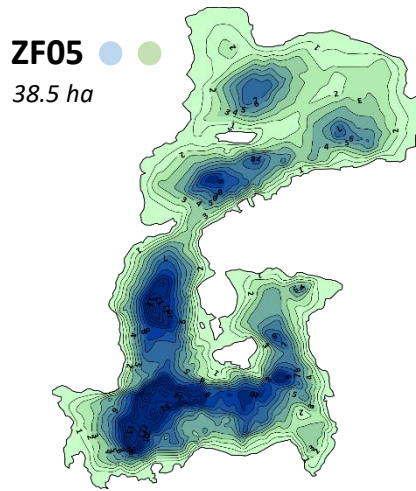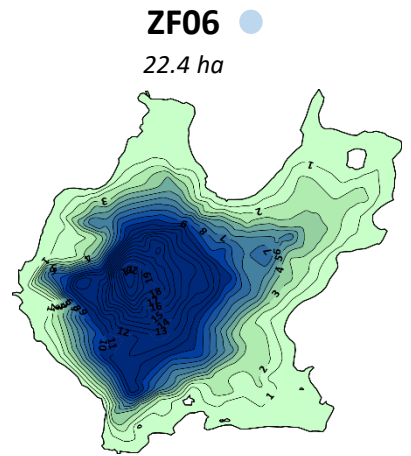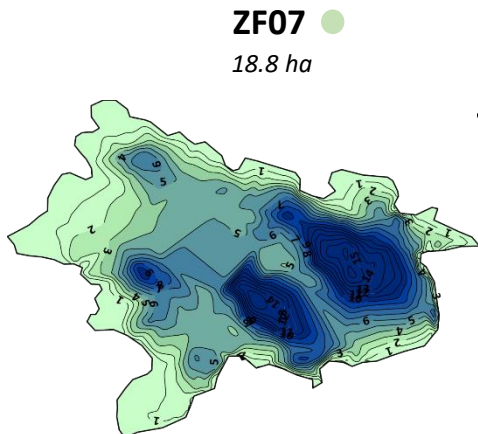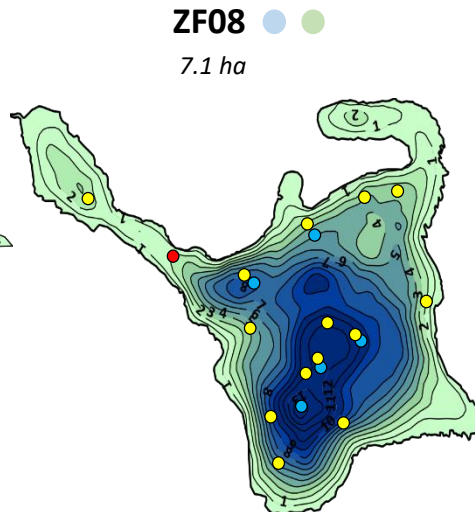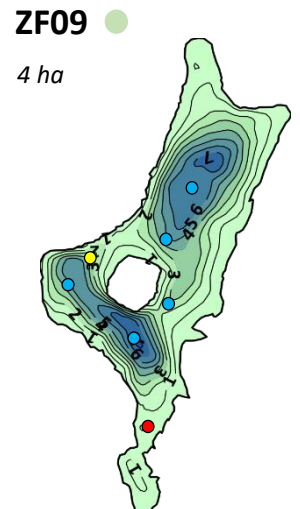

**ZF10** ● ●  
13.6 ha

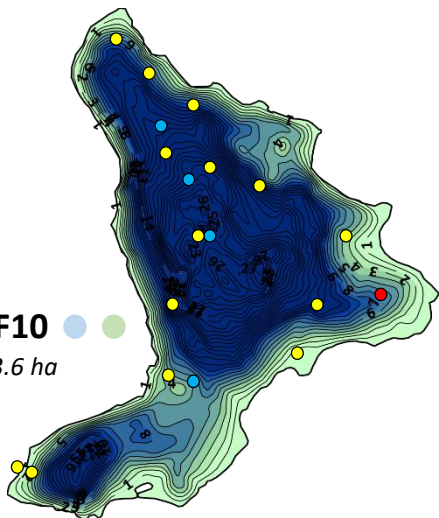

**ZF11** ● ●  
11.9 ha

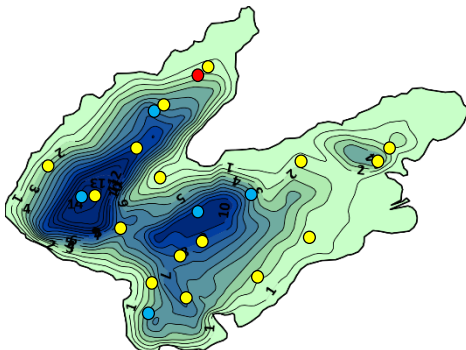

**ZF12** ● ●  
13.4 ha

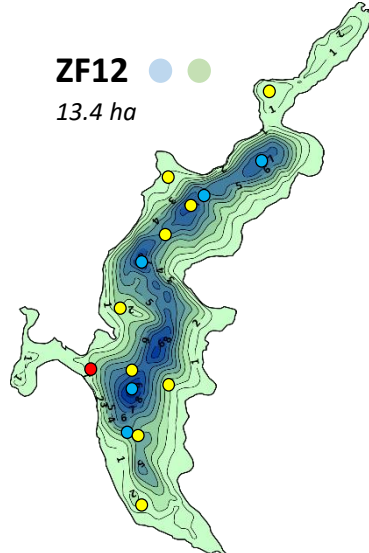

**ZF13** ● ●  
4.3 ha

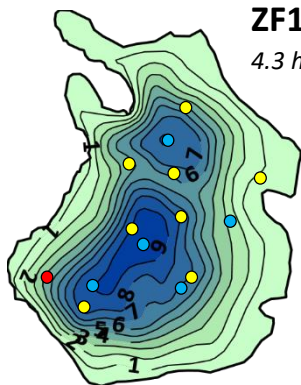

**ZF14** ● ●  
5 ha

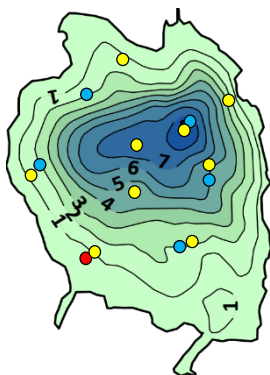

**ZF15** ● ●  
4.5 ha

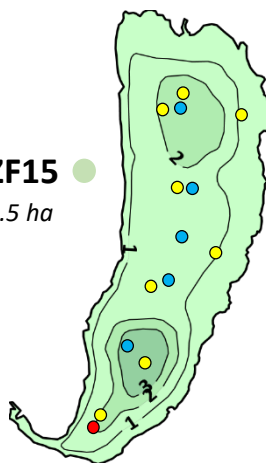

**ZF16** ● ●  
4.7 ha

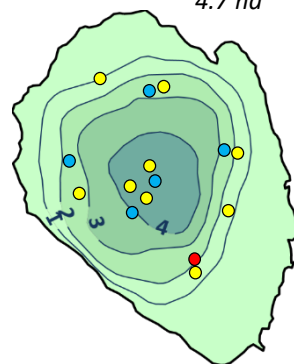

**ZF19** ● ●  
9.2 ha

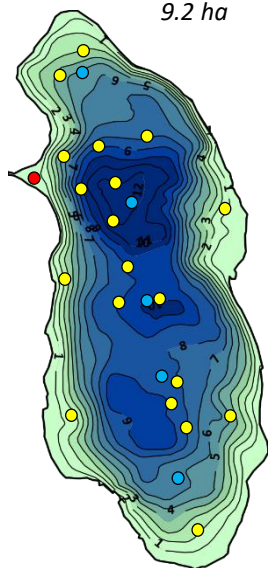

**ZF20** ● ●  
4.6 ha

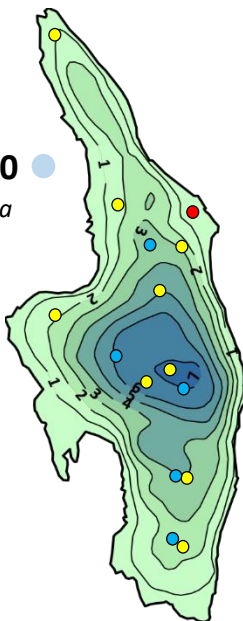

**ZF21** ● ●  
4.8 ha

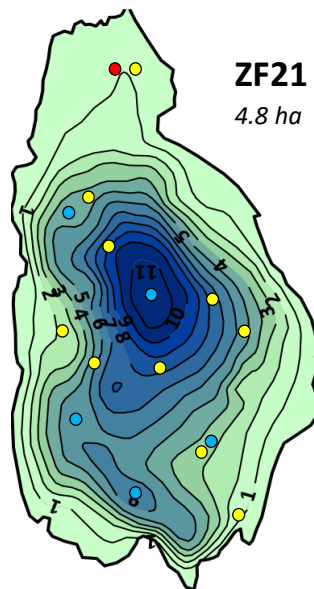

Supplement: S1 Fig — Locations of spatial replicate from in-lakes and outlet samples are displayed by blue and red circles respectively. Yellow circles showed the locations of nets for the gillnetting method. Locations for DNA sampling in 2016 field campaign were not recorded and performed accordingly to the description in the Materials and Methods section. (PDF) [file pone.0226638.s001.pdf]
